# Sustained LDL-C target attainment and recurrent ischemic cerebrovascular events after ischemic stroke: effect modification by large-artery disease

**DOI:** 10.3389/fneur.2026.1902614

**Published:** 2026-08-27

**Authors:** Xiaofeng Zhang, Di Niu, Yikang Li, Yuanyuan Wang, Ruizhi Chen, Guoping Xing

**Affiliations:** 1 School of Clinical Medicine, Shandong Second Medical University, Weifang, Shandong, China; 2 Department of Neurology, Weifang People's Hospital, Weifang, Shandong, China

**Keywords:** ischemic stroke, large-artery atherosclerosis, LDL-C target attainment, low-density lipoprotein cholesterol, recurrent cerebrovascular events, time-dependent Cox regression

## Abstract

**Background:**

In secondary prevention after ischemic stroke or TIA, LDL-C < 1.8 mmol/L is associated with a lower risk of recurrent vascular events. However, evidence remains limited in real-world patients with ischemic stroke, especially those with significant large-artery atherosclerotic stenosis. In particular, few studies have evaluated the impact of sustained LDL-C target attainment on recurrent ischemic cerebrovascular events by using LDL-C target attainment as a time-varying exposure.

**Methods:**

We retrospectively studied 933 patients with ischemic stroke who had baseline LDL-C levels ≥1.8 mmol/L at admission. Significant large-artery disease (LAD) was defined as ≥50% intracranial or extracranial atherosclerotic stenosis. LDL-C target attainment was defined as <1.8 mmol/L. The primary outcome was recurrent ischemic cerebrovascular events. Time-dependent Cox models evaluated the association between time-varying LDL-C target attainment and recurrence, and effect modification by LAD status was assessed. The proportion of follow-up time with LDL-C ≥ 1.8 mmol/L was additionally assessed.

**Results:**

Among 933 patients, 369 had LAD and 564 did not. During follow-up, 102 recurrent ischemic cerebrovascular events occurred. In the fully adjusted model, time-varying LDL-C target attainment was associated with lower recurrence risk [hazard ratio (HR) = 0.399, 95% confidence interval (CI): 0.269–0.593, *p* < 0.001]. This association was stronger in patients with LAD (HR = 0.282, 95% CI: 0.168–0.475, p < 0.001) than in those without LAD (HR = 0.530, 95% CI: 0.288–0.976; *p* = 0.042; P for interaction = 0.041). Each 10% increase in the proportion of follow-up time with LDL-C ≥ 1.8 mmol/L was associated with higher recurrence risk (HR = 1.189, 95% CI 1.127–1.254), with a stronger association in LAD patients (P for interaction = 0.008).

**Conclusion:**

Among patients with ischemic stroke who had baseline LDL-C levels ≥1.8 mmol/L at admission, sustained LDL-C target attainment was associated with a lower risk of recurrence after ischemic stroke. This association was particularly evident in patients with significant LAD. Sustained LDL-C control may support risk stratification and secondary prevention.

## 1 Introduction

Low-density lipoprotein cholesterol (LDL-C) is a key risk factor for atherosclerotic cardiovascular disease and an important therapeutic target for lipid-lowering therapy in clinical practice (1, 2). The SPARCL trial demonstrated that intensive statin therapy after stroke or transient ischemic attack reduced the risk of subsequent stroke and cardiovascular events (3). The CTT meta-analysis further showed that greater reductions in LDL-C were associated with greater reductions in the risk of major vascular events (4). On this basis, the TST trial supported an intensive LDL-C target of <1.8 mmol/L in patients with ischemic stroke or transient ischemic attack and evidence of atherosclerosis (5). Previous studies have mainly focused on the association between the magnitude of LDL-C reduction or fixed LDL-C targets and the risk of vascular events. However, in real-world practice, LDL-C levels fluctuate over time because of treatment intensification and variations in adherence (6). Therefore, this study used retrospective follow-up data from a real-world cohort of patients with ischemic stroke. All patients had baseline LDL-C levels ≥1.8 mmol/L at admission. According to guideline recommendations for secondary stroke prevention (7), LDL-C < 1.8 mmol/L was adopted as the target-attainment threshold. We assessed the association between time-varying LDL-C target attainment and recurrent ischemic cerebrovascular events. We also examined whether recurrence risk increased with a higher proportion of follow-up time spent above target. Effect modification by significant large-artery disease was further evaluated.

## 2 Methods

### 2.1 Study population

This retrospective study used pre-existing electronic medical records from patients admitted with ischemic stroke to the Department of Neurology, Weifang People’s Hospital, between July 1, 2024, and July 1, 2025. The exclusion criteria were as follows: (1) LDL-C level <1.8 mmol/L on admission; (2) no statin-based lipid-lowering therapy; (3) ischemic stroke not confirmed by brain magnetic resonance imaging; (4) no intracranial and extracranial vascular assessment by computed tomography angiography or magnetic resonance angiography, precluding evaluation of vascular stenosis; and (5) incomplete follow-up data, defined as absence of at least two routine outpatient follow-up records within 1 month and 1 to 3 months after discharge, or lack of corresponding lipid profile and liver function test results. This study focused on patients who had not achieved the recommended LDL-C target at admission and therefore represented a population requiring intensified lipid-lowering management. Accordingly, patients with baseline LDL-C < 1.8 mmol/L were not included in the primary analysis. After admission, all patients received atorvastatin or rosuvastatin at doses consistent with moderate-intensity lipid-lowering therapy, namely atorvastatin 10–20 mg/day or rosuvastatin 5–10 mg/day (8). The lipid-lowering regimen at the index admission was categorized as statin monotherapy or combination therapy. Combination therapy comprised a statin with ezetimibe and/or a proprotein convertase subtilisin/kexin type 9 (PCSK9) inhibitor. The study was approved by the Medical Ethics Committee of Weifang People’s Hospital (approval No. KYLL20240129-3). The requirement for informed consent was waived by the ethics committee because of the retrospective design and use of de-identified routinely collected clinical data.

### 2.2 Data collection

Baseline data were collected for all patients, including demographic characteristics (age and sex); vascular risk factors, including baseline blood pressure, smoking history, alcohol consumption, hypertension, hyperlipidemia, diabetes mellitus, history of coronary heart disease, atrial fibrillation, previous ischemic cerebrovascular events, and history of intracerebral hemorrhage; stroke territory; infarct laterality; culprit vessel; TOAST classification; National Institutes of Health Stroke Scale score on admission; baseline laboratory parameters, including total cholesterol, triglycerides, high-density lipoprotein cholesterol, baseline low-density lipoprotein cholesterol, and fasting blood glucose; and medication use.

### 2.3 Imaging assessment

Under the supervision of a neuroradiology consultant and two experienced clinicians, the severity of intracranial and extracranial large-artery atherosclerotic stenosis was assessed and graded according to the WASID criteria (9) and NASCET criteria (10), respectively, as follows: mild stenosis, <50%; moderate stenosis, 50–69%; and severe stenosis or occlusion, ≥70%.

Arterial lesions were further classified according to anatomical location. Extracranial arteries included the aortic arch, brachiocephalic artery, subclavian artery, common carotid artery, internal carotid artery segments 1 and 2, basilar artery, and vertebral artery segments 1 to 3. Intracranial arteries included internal carotid artery segments 3 to 7, vertebral artery segment 4, anterior cerebral artery, middle cerebral artery, and posterior cerebral artery.

### 2.4 Clinical outcomes and definitions

Clinical outcomes were followed for 9 months. The primary outcome was recurrent ischemic cerebrovascular events, and the safety outcome was intracerebral hemorrhage. The relevant definitions were as follows: (1) LAD group: patients with any intracranial or extracranial large-artery atherosclerotic stenosis, defined as luminal stenosis ≥50%; patients with stenosis <50% were classified into the non-LAD group (11). (2) Primary efficacy endpoint: recurrence of ischemic cerebrovascular events, including new-onset ischemic stroke or transient ischemic attack.

### 2.5 Statistical analysis

Continuous variables with a normal distribution were expressed as mean ± standard deviation and compared between groups using the independent-samples t test. Variables with a non-normal distribution were expressed as median and interquartile range and compared using the Mann–Whitney U test. Categorical variables were presented as frequencies and percentages, and between-group differences were assessed using the χ^2^ test or Fisher’s exact test, as appropriate. A two-sided *p* value <0.05 was considered statistically significant. LDL-C target attainment was selected as the primary exposure variable and was dichotomized using 1.8 mmol/L as the threshold. LDL-C < 1.8 mmol/L was defined as target attainment, whereas LDL-C ≥ 1.8 mmol/L was defined as non-attainment. Because LDL-C levels may change over the course of follow-up, LDL-C target attainment status was incorporated into Cox proportional hazards models as a time-dependent covariate. The primary outcome was recurrent ischemic cerebrovascular events, and intracerebral hemorrhage was treated as a censoring event in the analysis. Cox regression models were constructed to evaluate the association between time-varying LDL-C target attainment and the risk of recurrent ischemic cerebrovascular events. Model 1 was unadjusted. Model 2 was adjusted for age and sex. Model 3 was further adjusted for TOAST classification, NIHSS score on admission, hypertension, smoking, and lipid-lowering treatment regimen on admission. The proportional hazards assumption was assessed using Schoenfeld residuals. Subgroup analyses were performed according to age, sex, NIHSS score on admission, smoking, hypertension, and LAD status. Potential effect modification was assessed by adding product terms between LDL-C target attainment status and subgroup variables to Model 3. To assess the robustness of the main findings, we repeated the time-dependent Cox regression analyses after excluding patients who experienced recurrent events within 60 days of follow-up. We also performed time-stratified sensitivity analyses. The overall follow-up period was divided into two intervals: 0–3 months and 3–9 months. Time-dependent Cox regression models were then fitted separately within each interval. The covariate adjustment strategy was consistent with that used in the main analysis. As a supplementary analysis, the proportion of follow-up time during which each patient had LDL-C ≥ 1.8 mmol/L was calculated and included in the Cox proportional hazards model as a continuous variable. A product term between the proportion of follow-up time with LDL-C ≥ 1.8 mmol/L and LAD status was added to Model 3 to assess effect modification by LAD status.

## 3 Results

### 3.1 Baseline characteristics and clinical outcomes

**Figure 1:**
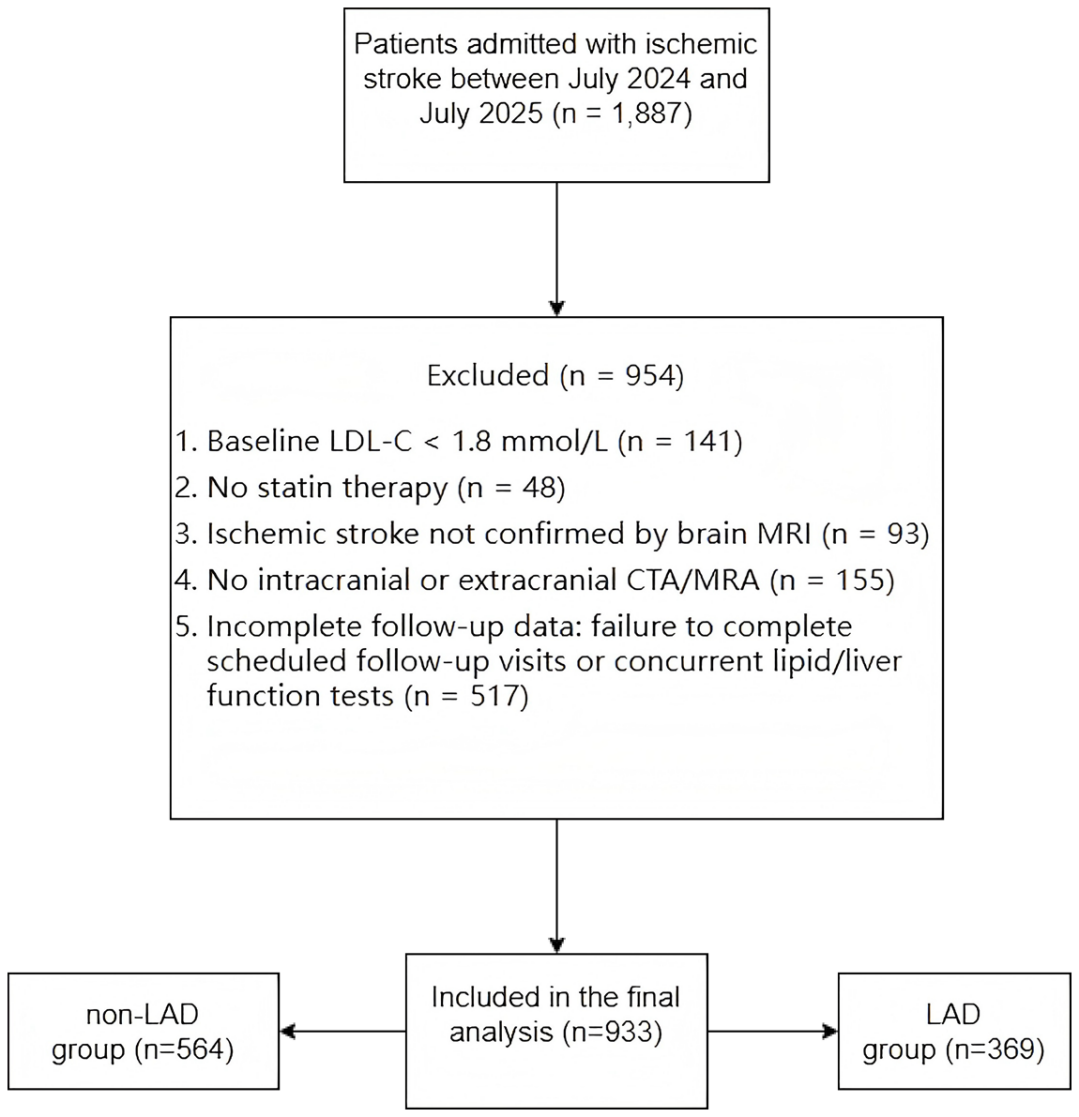
Patient selection flowchart. Flowchart of patient selection. A total of 1,887 patients admitted with ischemic stroke between July 2024 and July 2025 were screened. Patients were excluded for baseline LDL-C < 1.8 mmol/L, no statin therapy, ischemic stroke not confirmed by brain MRI, absence of intracranial or extracranial CTA/MRA, or incomplete follow-up data. Finally, 933 patients were included in the analysis, including 564 patients in the non-LAD group and 369 patients in the LAD group.

A total of 933 patients with ischemic stroke were included in this study, including 369 patients in the large-artery disease (LAD) group and 564 patients in the non-LAD group (Figure 1). The mean age of the overall population was 64.04 ± 10.97 years, and 617 patients were male, accounting for 66.1%. A previous ischemic cerebrovascular event was documented in 208 patients (22.3%), with similar proportions in the LAD and non-LAD groups (23.6% vs. 21.5%, *p* = 0.496). Compared with patients in the non-LAD group, those in the LAD group were older (65.20 ± 10.39 years vs. 63.28 ± 11.27 years, *p* = 0.008), had a higher prevalence of hypertension (82.1% vs. 74.1%, *p* = 0.006), and had higher NIHSS scores on admission (5.06 ± 4.58 vs. 3.32 ± 3.29, *p* < 0.001), suggesting greater baseline clinical severity in the LAD group. The proportion of anterior circulation stroke was also higher in the LAD group than in the non-LAD group (62.6% vs. 53.4%), with a statistically significant difference in stroke territory between the two groups (*p* = 0.007). TOAST classification differed significantly between groups, with a markedly higher proportion of large-artery atherosclerosis in the LAD group (85.6% vs. 37.1%, *p* < 0.001). Baseline LDL-C levels were higher in the LAD group than in the non-LAD group (3.11 ± 0.81 mmol/L vs. 2.92 ± 0.69 mmol/L, *p* < 0.001). The proportion of patients receiving a statin combined with ezetimibe and/or a PCSK9 inhibitor was higher in the LAD group than in the non-LAD group, although the difference did not reach statistical significance (68.3% vs. 61.9%, *p* = 0.054) (Table 1).

**Table 1:** Baseline characteristics of the study population.

Characteristic	Overall (*n* = 933)	non-LAD group (*n* = 564)	LAD group (*n* = 369)	*P* value
Demographics				
Age, years	64.04 ± 10.97	63.28 ± 11.27	65.20 ± 10.39	0.008
Male	617 (66.1%)	366 (64.9%)	251 (68.0%)	0.359
Baseline blood pressure				
Systolic blood pressure, mmHg	154.62 ± 21.86	153.52 ± 20.33	156.29 ± 23.94	0.068
Diastolic blood pressure, mmHg	90.13 ± 13.98	89.51 ± 13.62	91.08 ± 14.48	0.099
Vascular risk factors				
Smoking	428 (45.9%)	260 (46.1%)	168 (45.5%)	0.917
Hypertension	721 (77.3%)	418 (74.1%)	303 (82.1%)	0.006
Hyperlipidemia	115 (12.3%)	71 (12.6%)	44 (11.9%)	0.841
Diabetes mellitus	322 (34.5%)	184 (32.6%)	138 (37.4%)	0.153
Coronary heart disease	102 (10.9%)	63 (11.2%)	39 (10.6%)	0.857
Atrial fibrillation	74 (7.9%)	46 (8.2%)	28 (7.6%)	0.849
Prior ischemic cerebrovascular event	208 (22.3%)	121 (21.5%)	87 (23.6%)	0.496
History of intracerebral hemorrhage	25 (2.7%)	16 (2.8%)	9 (2.4%)	0.872
Stroke territory				0.007
Anterior circulation	532 (57.0%)	301 (53.4%)	231 (62.6%)	
Posterior circulation	401 (43.0%)	263 (46.6%)	138 (37.4%)	
Infarct laterality				0.098
Left	456 (48.9%)	278 (49.3%)	178 (48.2%)	
Right	427 (45.8%)	263 (46.6%)	164 (44.4%)	
Bilateral	50 (5.4%)	23 (4.1%)	27 (7.3%)	
Responsible artery				0.064
Anterior cerebral artery	78 (8.4%)	56 (9.9%)	22 (6.0%)	
Middle cerebral artery	343 (36.8%)	206 (36.5%)	137 (37.1%)	
Internal carotid artery	148 (15.9%)	77 (13.7%)	71 (19.2%)	
Posterior cerebral artery	149 (16.0%)	92 (16.3%)	57 (15.4%)	
Vertebrobasilar artery	215 (23.0%)	133 (23.6%)	82 (22.2%)	
TOAST classification				<0.001
Large-artery atherosclerosis	525 (56.3%)	209 (37.1%)	316 (85.6%)	
Small-vessel occlusion	249 (26.7%)	230 (40.8%)	19 (5.1%)	
Cardioembolic and other etiologies	159 (17.0%)	125 (22.2%)	34 (9.2%)	
NIHSS score	4.00 ± 3.94	3.32 ± 3.29	5.06 ± 4.58	<0.001
Baseline laboratory tests				
Total cholesterol, mmol/L	4.57 ± 0.95	4.56 ± 0.89	4.58 ± 1.04	0.680
Triglycerides, mmol/L	1.51 ± 0.95	1.53 ± 0.95	1.46 ± 0.97	0.264
High-density lipoprotein cholesterol, mmol/L	1.12 ± 0.28	1.12 ± 0.26	1.12 ± 0.30	0.965
Low-density lipoprotein cholesterol, mmol/L	3.00 ± 0.74	2.92 ± 0.69	3.11 ± 0.81	<0.001
Fasting glucose, mmol/L	6.66 ± 2.56	6.54 ± 2.48	6.86 ± 2.68	0.068
Lipid-lowering therapy				0.054
Statin monotherapy	332 (35.6%)	215 (38.1%)	117 (31.7%)	
Combination with non-statin lipid-lowering therapy	601 (64.4%)	349 (61.9%)	252 (68.3%)	
Antiplatelet therapy				0.076
Single antiplatelet therapy	310 (33.2%)	200 (35.5%)	110 (29.8%)	
Dual antiplatelet therapy	549 (58.8%)	319 (56.6%)	230 (62.3%)	
Clinical outcomes				
Ischemic cerebrovascular event	102 (10.9%)	49 (8.7%)	53 (14.4%)	0.009
Ischemic stroke	90 (9.6%)	44 (7.8%)	46 (12.5%)	0.025
Transient ischemic attack	12 (1.3%)	5 (0.9%)	7 (1.9%)	0.297
Intracerebral hemorrhage	6(0.6%)	5(0.9%)	1(0.3%)	0.412

During follow-up, 102 recurrent ischemic cerebrovascular events occurred, accounting for 10.9% of the overall cohort. The incidence of ischemic cerebrovascular events was higher in the LAD group than in the non-LAD group (14.4% vs. 8.7%, *p* = 0.009). Similarly, the recurrence rate of ischemic stroke was higher in the LAD group (12.5% vs. 7.8%, *p* = 0.025). Intracerebral hemorrhage occurred in 6 patients (0.6%) overall, including 5 patients in the non-LAD group (0.9%) and 1 patient in the LAD group (0.3%), with no significant difference between groups (*p* = 0.412) (Table 1). Because of the limited number of intracerebral hemorrhage events, no further regression analysis was performed for this safety outcome.

### 3.2 Association between LDL-C target attainment and the risk of recurrent ischemic cerebrovascular events

In patients with ischemic stroke and baseline LDL-C levels ≥1.8 mmol/L, time-varying LDL-C target attainment was associated with a reduced risk of recurrent ischemic cerebrovascular events. In the unadjusted model, patients who achieved the LDL-C target had a lower risk of recurrence than those who did not, with a hazard ratio (HR) of 0.38 [95% confidence interval (CI): 0.26–0.57, *p* < 0.001]. After adjustment for age and sex, this association remained stable, with an HR of 0.38 (95% CI: 0.25–0.57, *p* < 0.001). After further adjustment for NIHSS score on admission, TOAST classification, and other covariates, the association remained statistically significant, with an HR of 0.399 (95% CI: 0.27–0.59, *p* < 0.001). In the non-LAD group, LDL-C target attainment was also associated with a lower risk of recurrence. In Model 3, this association remained statistically significant, with an HR of 0.53 (95% CI: 0.29–0.98, *p* = 0.0417). The protective association of time-varying LDL-C target attainment was more pronounced in the LAD group, with an HR of 0.28 (95% CI: 0.17–0.48, *p* < 0.001) (Table 2). The proportion of follow-up time with LDL-C ≥ 1.8 mmol/L was significantly and positively associated with the risk of recurrent ischemic cerebrovascular events. This association was statistically significant in both the non-LAD and LAD groups, with HRs of 1.14 (95% CI: 1.06–1.22, *p* < 0.001) and 1.29 (95% CI: 1.18–1.40, *p* < 0.001), respectively. Evidence of effect modification by LAD status was observed for the association between the proportion of follow-up time with LDL-C ≥ 1.8 mmol/L and recurrence risk (P for interaction = 0.008) (Table 3).

**Table 2:** Association between time-dependent LDL-C target attainment and recurrent ischemic cerebrovascular events.

Subgroup	Model	HR (95% CI)	*P* value	P for interaction
Overall	Model 1	0.380 (0.255–0.567)	<0.001	
	Model 2	0.380 (0.254–0.568)	<0.001	
	Model 3	0.399 (0.269–0.593)	<0.001	
Non-LAD	Model 1	0.525 (0.297–0.929)	0.0269	
	Model 2	0.531 (0.297–0.949)	0.0326	
	Model 3	0.530 (0.288–0.976)	0.0417	
LAD	Model 1	0.234 (0.133–0.410)	<0.001	
	Model 2	0.235 (0.135–0.409)	<0.001	
	Model 3	0.282 (0.168–0.475)	<0.001	0.0414

**Table 3:** Association between the nontarget LDL-C time proportion and recurrent ischemic cerebrovascular events.

Group	N	Events	HR (95% CI)	*P* value	P for interaction
Overall	933	102	1.189 (1.127–1.254)	<0.001	
Non-LAD	564	49	1.137 (1.057–1.224)	<0.001	
LAD	369	53	1.288 (1.183–1.402)	<0.001	0.008

### 3.3 Subgroup analyses and assessment of effect modification

**Figure 2:**
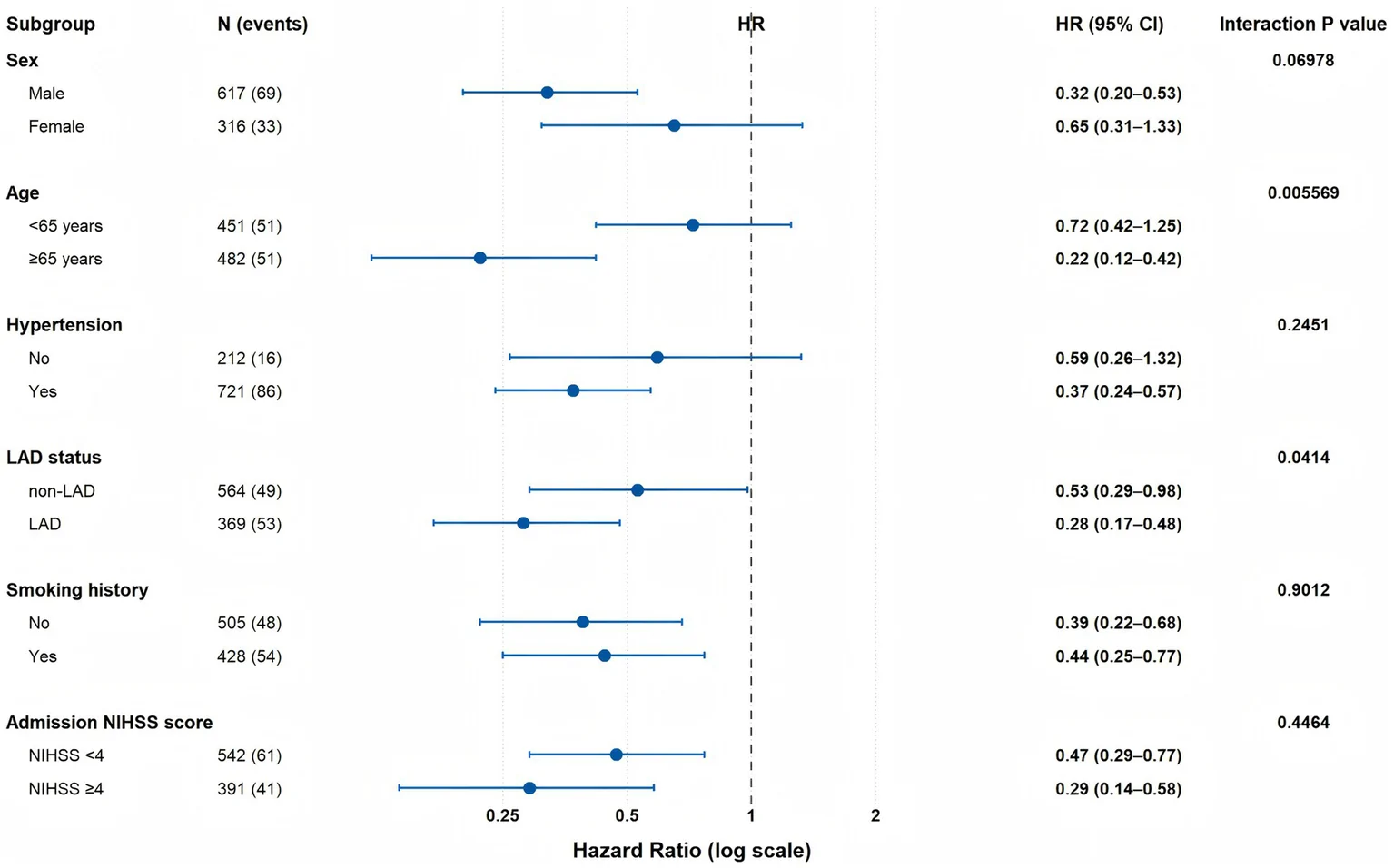
Subgroup analysis of LDL-C target attainment and recurrence risk. Subgroup analysis of the association between LDL-C target attainment and recurrent ischemic cerebrovascular events. Hazard ratios were estimated using time-dependent Cox regression, comparing LDL-C < 1.8 mmol/L with LDL-C ≥ 1.8 mmol/L. Subgroups included sex, age, hypertension, LAD status, smoking history, and admission NIHSS score. Values are shown as hazard ratios with 95% confidence intervals. *p* values for interaction were calculated by adding product terms between LDL-C target attainment and subgroup variables to the adjusted model.

Subgroup analyses further showed that the association between LDL-C target attainment and lower recurrence risk was generally consistent across clinically relevant subgroups. Evidence of effect modification was observed for age and LAD status. Among patients aged ≥65 years, LDL-C target attainment was associated with a significantly lower recurrence risk (HR = 0.22, 95% CI: 0.11–0.42, *p* < 0.001), whereas the association did not reach statistical significance among patients aged <65 years (HR = 0.72, 95% CI: 0.42–1.25, *p* = 0.249; P for interaction = 0.006), suggesting a stronger protective association in older patients. In the LAD group, LDL-C target attainment was associated with a marked reduction in recurrence risk (HR = 0.28, 95% CI: 0.17–0.47), while the association was weaker but remained statistically significant in the non-LAD group (HR = 0.53, 95% CI: 0.29–0.98; P for interaction = 0.041). These findings suggest that the protective association of LDL-C target attainment may be more pronounced in patients with LAD. Significant associations were also observed in male patients (HR = 0.32, 95% CI: 0.20–0.53), patients with hypertension (HR = 0.37, 95% CI: 0.24–0.57), smokers (HR = 0.44, 95% CI: 0.25–0.77), and patients with admission NIHSS score ≥4 (HR = 0.29, 95% CI: 0.14–0.58). However, evidence of effect modification was not observed for these subgroups (Figure 2 and Supplementary Table 1).

### 3.4 Sensitivity analysis and proportional hazards assumption

After excluding patients who experienced recurrence within 60 days, 920 patients remained in the analysis, with 89 recurrent ischemic cerebrovascular events. In the fully adjusted model, time-varying LDL-C target attainment remained significantly associated with a lower risk of recurrence in patients with ischemic stroke and baseline LDL-C levels ≥1.8 mmol/L (HR = 0.39, 95% CI: 0.25–0.59, *p* < 0.001). This association remained significant in the LAD group (HR = 0.25, 95% CI: 0.14–0.45, p < 0.001). In the non-LAD group, the association was attenuated and did not reach statistical significance, although the direction of effect remained consistent (HR = 0.54, 95% CI: 0.29–1.02, *p* = 0.057). This finding suggests a possible trend that requires further validation in larger studies with more events. Evidence of effect modification by LAD status remained statistically significant (P for interaction = 0.034) (Supplementary Table 2).

During the first 3 months of follow-up, the association between LDL-C target attainment and recurrent ischemic cerebrovascular events was not statistically significant. In patients with ischemic stroke and baseline LDL-C levels ≥1.8 mmol/L, the HR was 0.740 (95% CI: 0.338–1.620, *p* = 0.452) (Supplementary Table 3). During the 3-to 9-month follow-up period, LDL-C target attainment remained significantly associated with a lower risk of recurrence (HR = 0.355, 95% CI: 0.226–0.556, *p* < 0.001). The corresponding HR was 0.499 in the non-LAD group (95% CI: 0.258–0.966, *p* = 0.039) and 0.230 in the LAD group (95% CI: 0.126–0.421, p < 0.001). Evidence of effect modification by LAD status was also observed (P for interaction = 0.042) (Supplementary Table 4).

The proportional hazards assumption was assessed using Schoenfeld residuals, and no significant violation was observed for LDL-C target attainment or the adjusted covariates (global test, *p* > 0.05) (Supplementary Figure 1).

## 4 Discussion

This real-world cohort comprised patients with ischemic stroke and baseline LDL-C ≥ 1.8 mmol/L. An LDL-C level <1.8 mmol/L, modeled as a time-varying exposure, was associated with a lower risk of recurrent ischemic cerebrovascular events. Conversely, a greater proportion of follow-up time with LDL-C ≥ 1.8 mmol/L was associated with a higher risk of recurrence. These associations were more pronounced among patients with LAD. A key strength and novel feature of this study is the evaluation of serial LDL-C status (<1.8 versus ≥1.8 mmol/L) as a dynamic, time-dependent exposure rather than relying on a single baseline measurement. From a clinical perspective, the findings highlight the potential relevance of maintaining LDL-C < 1.8 mmol/L over time, particularly in patients with significant large-artery atherosclerotic stenosis.

These findings extend those of previous longitudinal studies investigating LDL-C after ischemic stroke. In a Chinese cohort, Lau et al. found that a mean follow-up LDL-C level <1.8 mmol/L was associated with lower risks of recurrent stroke and major adverse cardiovascular events in patients with and without LAD (11). That study used mean follow-up LDL-C to summarize overall exposure. By contrast, the present time-dependent analysis captured changes between LDL-C < 1.8 and ≥1.8 mmol/L throughout follow-up. It also preserved the temporal ordering between serial LDL-C assessments and subsequent recurrent events. Longitudinal lipid measurements have also been examined in relation to ischemic stroke risk in the Northern Manhattan Study (12).

Notably, lipid-related risk after stroke may not be fully captured by LDL-C alone. In patients with ischemic stroke, higher Lp(a) levels have been associated with an increased risk of recurrent vascular events and post-stroke cognitive impairment (13, 14). These findings suggest that Lp(a) may contribute to adverse outcomes and residual risk after stroke. Future studies integrating LDL-C, Lp(a), and other ApoB-containing lipoprotein measures may provide a more comprehensive understanding of the sources of residual risk among patients who achieve LDL-C targets.

The stronger association observed in patients with LAD is biologically plausible. LDL-C is a key driver of the development and progression of atherosclerosis. Mechanistically, LDL-C and other apolipoprotein B-containing lipoproteins can enter and become retained in the arterial intima. These lipoproteins may then undergo modification and induce local inflammatory responses. This process promotes macrophage foam cell formation, expansion of the lipid-rich necrotic core, and plaque progression (15, 16). As plaque burden increases, a larger necrotic core, a thin fibrous cap, and inflammatory cell infiltration may collectively increase the risk of plaque rupture and thrombosis (17). Patients with LAD generally have a higher local atherosclerotic plaque burden. Previous high-resolution vessel wall MRI studies have shown that greater plaque burden is associated with an increased risk of stroke recurrence (18, 19). A higher proportion of follow-up time with LDL-C ≥ 1.8 mmol/L may further aggravate lipid deposition and inflammatory activation within stenotic arterial segments. These changes may promote plaque progression and increase plaque vulnerability. In addition, abnormal local shear stress in stenotic arteries may worsen endothelial dysfunction in patients with LAD. It may also increase vascular permeability. These changes may facilitate LDL entry into the intima and promote LDL oxidation. This process may further amplify local inflammation and accelerate plaque progression (20, 21). LDL-C lowering may directly reduce plaque lipid-core volume and increase fibrous-cap thickness, thereby stabilizing vulnerable plaques. Statins and PCSK9 inhibitors may also improve endothelial function and suppress inflammation through pleiotropic or potentially lipid-independent vascular effects (22–24). Therefore, in clinical practice, intensive lipid-lowering therapy should be initiated early. This approach may help patients achieve LDL-C targets more rapidly and reduce the duration of LDL-C ≥ 1.8 mmol/L. It may be particularly important for patients with ischemic stroke and significant large-artery stenosis. In addition, polyphenol-rich diets may improve lipid metabolism by reducing oxidative stress, regulating lipid synthesis, and promoting cholesterol transport. These diets may therefore complement pharmacological lipid-lowering therapy (25).

In this study, age subgroup analysis suggested possible effect modification of the association between LDL-C non-attainment and recurrence risk. This association appeared to be more pronounced in patients aged ≥65 years. However, the association did not reach statistical significance in patients aged <65 years. This may be related to the limited number of events or insufficient statistical power. Therefore, this finding should be interpreted with caution. Older patients usually have a longer duration of cumulative exposure to vascular risk factors and a higher baseline atherosclerotic burden. In addition, vascular aging is characterized by increased arterial stiffness, endothelial dysfunction, vascular remodeling, oxidative stress, and chronic low-grade inflammation (26). These changes may increase susceptibility to ischemic stroke in older adults and may impair the compensatory capacity of the cerebral vasculature in response to ischemic and metabolic stress (27). Therefore, in patients aged ≥65 years with ischemic stroke, lipid-lowering regimens should be adjusted in a timely manner through serial lipid monitoring. This approach may help achieve LDL-C targets earlier and further reduce the risk of ischemic recurrence.

Higher NIHSS scores have been independently associated with an increased risk of recurrent stroke and major vascular events (28). A recent study further showed that NIHSS scores were associated with the systemic inflammatory response index and high-sensitivity C-reactive protein and suggested that NIHSS might partially mediate their associations with functional outcomes (29). These findings suggest that the NIHSS is not only a quantitative measure of stroke severity but may also reflect inflammatory status and recurrent vascular risk. However, inflammatory biomarkers were not systematically available in this cohort, precluding direct evaluation of this hypothesis. Although LDL-C target attainment was associated with a lower recurrence risk in the NIHSS ≥4 subgroup, there was no evidence of effect modification by NIHSS score (P for interaction = 0.446). Therefore, this finding should not be interpreted as a distinct subgroup-specific effect. Similarly, no evidence of effect modification was observed by sex, hypertension, or smoking. Therefore, while strengthening LDL-C target-oriented management, comprehensive risk factor control, including blood pressure management and smoking cessation, should also be implemented. In addition, psychological stress may promote the progression of atherosclerosis through endothelial dysfunction, inflammatory activation, and reduced treatment adherence. Its potential impact on secondary stroke prevention also warrants attention (30).

This study has several limitations. First, the cohort was restricted to patients with baseline LDL-C ≥ 1.8 mmol/L who received statin-based therapy, representing a selected population requiring intensified lipid management. Accordingly, the findings may not be generalizable to patients with LDL-C < 1.8 mmol/L at admission, including those whose LDL-C subsequently increased to ≥1.8 mmol/L, those not receiving pharmacological lipid-lowering therapy, or unselected stroke populations in other healthcare settings. Confounding by indication may also have occurred because baseline vascular risk may have influenced both the intensity of lipid-lowering therapy and the risk of recurrence. Second, the single-center retrospective design remained susceptible to residual confounding despite multivariable adjustment. A history of ischemic cerebrovascular events, present in 22.3% of participants and clinically relevant to recurrence risk, was not included in the fully adjusted model. Body mass index was not systematically documented, and CRP, hs-CRP, and other inflammatory indices were not routinely measured. Consequently, residual confounding related to pre-existing cerebrovascular disease burden, adiposity, and inflammatory status cannot be excluded. Third, lipid testing depended on outpatient follow-up, while changes in drug dose, combination therapy, treatment adherence, diet, and lifestyle were incompletely recorded. Consequently, LDL-C fluctuations and factors affecting target attainment may not have been fully captured. Fourth, the 9-month follow-up did not permit assessment of long-term LDL-C exposure. Early events may also be influenced by acute plaque instability and the timing of secondary prevention therapy. The association was not statistically significant during the first 3 months but was observed during months 3–9; longer follow-up is therefore required. Finally, the limited number of events in some subgroups may have reduced statistical power and precision. Therefore, findings regarding effect modification by LAD and age should be interpreted cautiously and validated in larger multicenter prospective studies.

## 5 Conclusion

Sustained LDL-C target attainment was associated with a lower risk of recurrent ischemic cerebrovascular events during the 9-month follow-up among patients with ischemic stroke and baseline LDL-C ≥ 1.8 mmol/L, particularly in those with LAD. The age-related subgroup finding requires further validation.

## Data Availability

The raw data supporting the conclusions of this article will be made available by the authors, without undue reservation.

## Supplementary material

SUPPLEMENTARY FIGURE 1 Schoenfeld residuals for proportional hazards assumption. Scaled Schoenfeld residual plots for evaluating the proportional hazards assumption in the multivariable Cox model. Each panel shows residuals over transformed time for LDL-C status and adjusted covariates, including sex, age, smoking history, admission NIHSS score, TOAST classification, hypertension, and combination lipid-lowering therapy. No significant violation of the proportional hazards assumption was observed based on Schoenfeld residual tests.

## References

[1] Ference BA Ginsberg HN Graham I Ray KK Packard CJ Bruckert E . Low-density lipoproteins cause atherosclerotic cardiovascular disease. 1. Evidence from genetic, epidemiologic, and clinical studies. A consensus statement from the European atherosclerosis society consensus panel. Eur Heart J. (2017) 38:2459–72. doi: 10.1093/eurheartj/ehx144, 28444290 PMC5837225

[2] Grundy SM Stone NJ Bailey AL Beam C Birtcher KK Blumenthal RS . AHA/ACC/AACVPR/AAPA/ABC/ACPM/ADA/AGS/APhA/ASPC/NLA/PCNA guideline on the management of blood cholesterol: a report of the American College of Cardiology/American Heart Association task force on clinical practice guidelines. Circulation. (2018) 139:e1082–143. doi: 10.1161/CIR.0000000000000625 30586774 PMC7403606

[3] Amarenco P Bogousslavsky J Callahan A Goldstein LB Hennerici M Rudolph AE . High-dose atorvastatin after stroke or transient ischemic attack. N Engl J Med. (2006) 355:549–59. doi: 10.1056/NEJMoa061894 16899775

[4] Cholesterol Treatment Trialists’ (CTT) Collaboration, Baigent C Blackwell L Emberson J Holland LE Reith C . Efficacy and safety of more intensive lowering of LDL cholesterol: a meta-analysis of data from 170,000 participants in 26 randomised trials. Lancet. (2010) 376:1670–81. 21067804 10.1016/S0140-6736(10)61350-5 PMC2988224

[5] Amarenco P Kim JS Labreuche J Charles H Abtan J Béjot Y . A comparison of two LDL cholesterol targets after ischemic stroke. N Engl J Med. (2020) 382:9–19. doi: 10.1056/NEJMoa1910355, 31738483

[6] Fang H Gao Q Lee ML Hsu W Tan NC. LDL-cholesterol change and goal attainment following statin intensity titration among Asians in primary care: a retrospective cohort study. Lipids Health Dis. (2021) 20:2. doi: 10.1186/s12944-020-01427-z, 33407522 PMC7788928

[7] Kleindorfer DO Towfighi A Chaturvedi S Cockroft KM Gutierrez J Lombardi-Hill D . 2021 guideline for the prevention of stroke in patients with stroke and transient ischemic attack: a guideline from the American Heart Association/American Stroke Association. Stroke. (2021) 52:e364–467. doi: 10.1161/STR.0000000000000375, 34024117

[8] Mach F Koskinas KC Roeters van Lennep JE Tokgözoğlu L Badimon L Baigent C . Focused update of the 2019 ESC/EAS guidelines for the management of dyslipidaemias. Eur Heart J. (2025) 46:4359–78. doi: 10.1093/eurheartj/ehaf190 40878289

[9] Chimowitz MI Lynn MJ Howlett-Smith H Stern BJ Hertzberg VS Frankel MR . Comparison of warfarin and aspirin for symptomatic intracranial arterial stenosis. N Engl J Med. (2005) 352:1305–16. doi: 10.1056/NEJMoa043033, 15800226

[10] North American Symptomatic Carotid Endarterectomy Trial Collaborators, Barnett H Taylor DW Haynes RB Sackett DL Peerless SJ . Beneficial effect of carotid endarterectomy in symptomatic patients with high-grade carotid stenosis. N Engl J Med. (1991) 325:445–53. doi: 10.1056/NEJM199108153250701, 1852179

[11] Lau KK Chua BJ Ng A Leung IYH Wong YK Chan AHY . Low-density lipoprotein cholesterol and risk of recurrent vascular events in Chinese patients with ischemic stroke with and without significant atherosclerosis. J Am Heart Assoc. (2021) 10:e021855. doi: 10.1161/JAHA.121.021855, 34369170 PMC8475056

[12] Willey JZ Xu Q Boden-Albala B Paik MC Moon YP Sacco RL . Lipid profile components and risk of ischemic stroke: the northern Manhattan study (NOMAS). Arch Neurol. (2009) 66:1400–6. doi: 10.1001/archneurol.2009.210, 19901173 PMC2830863

[13] Lange KS Nave AH Liman TG Grittner U Endres M Ebinger M. Lipoprotein(a) levels and recurrent vascular events after first ischemic stroke. Stroke. (2017) 48:36–42. doi: 10.1161/STROKEAHA.116.014436, 27856951

[14] Zhang T Xu R Bao L Zhang W. The correlation between lipoprotein(a) and cognitive impairment in patients with ischemic stroke. Br J Hosp Med. (2025) 86:1–17. doi: 10.12968/hmed.2024.0711, 40847982

[15] Borén J Chapman MJ Krauss RM Packard CJ Bentzon JF Binder CJ . Low-density lipoproteins cause atherosclerotic cardiovascular disease: pathophysiological, genetic, and therapeutic insights: a consensus statement from the European atherosclerosis society consensus panel. Eur Heart J. (2020) 41:2313–30. doi: 10.1093/eurheartj/ehz962, 32052833 PMC7308544

[16] Björkegren J Lusis AJ. Atherosclerosis: recent developments. Cell. (2022) 185:1630–45. doi: 10.1016/j.cell.2022.04.004, 35504280 PMC9119695

[17] Kawai K Kawakami R Finn AV Virmani R. Differences in stable and unstable atherosclerotic plaque. Arterioscler Thromb Vasc Biol. (2024) 44:1474–84. doi: 10.1161/ATVBAHA.124.319396, 38924440

[18] Ran Y Wang Y Zhu M Wu X Malhotra A Lei X . Higher plaque burden of middle cerebral artery is associated with recurrent ischemic stroke: a quantitative magnetic resonance imaging study. Stroke. (2020) 51:659–62. doi: 10.1161/STROKEAHA.119.028405, 31856694

[19] Lv Y Ma X Zhao W Ju J Yan P Li S . Association of plaque characteristics with long-term stroke recurrence in patients with intracranial atherosclerotic disease: a 3D high-resolution MRI-based cohort study. Eur Radiol. (2024) 34:3022–31. doi: 10.1007/s00330-023-10278-y, 37870623 PMC11126465

[20] Gimbrone MA García-Cardeña G. Endothelial cell dysfunction and the pathobiology of atherosclerosis. Circ Res. (2016) 118:620–36. doi: 10.1161/CIRCRESAHA.115.306301, 26892962 PMC4762052

[21] Mundi S Massaro M Scoditti E Carluccio MA van Hinsbergh VWM Iruela-Arispe ML . Endothelial permeability, LDL deposition, and cardiovascular risk factors-a review. Cardiovasc Res. (2018) 114:35–52. doi: 10.1093/cvr/cvx226, 29228169 PMC7729208

[22] Oesterle A Laufs U Liao JK. Pleiotropic effects of statins on the cardiovascular system. Circ Res. (2017) 120:229–43. doi: 10.1161/CIRCRESAHA.116.308537, 28057795 PMC5467317

[23] Räber L Ueki Y Otsuka T Losdat S Häner JD Lonborg J . Effect of Alirocumab added to high-intensity statin therapy on coronary atherosclerosis in patients with acute myocardial infarction: the PACMAN-AMI randomized clinical trial. JAMA. (2022) 327:1771–81. doi: 10.1001/jama.2022.5218, 35368058 PMC8978048

[24] Momtazi-Borojeni AA Sabouri-Rad S Gotto AM Pirro M Banach M Awan Z . PCSK9 and inflammation: a review of experimental and clinical evidence. Eur Heart J Cardiovasc Pharmacother. (2019) 5:237–45. doi: 10.1093/ehjcvp/pvz022, 31236571

[25] Scavuzzi BM Dichi I. Effects of a diet rich in polyphenols on lipid metabolism: an updated narrative review. Heart Mind. (2025) 9:264–76. doi: 10.4103/hm.HM-D-24-00106

[26] Li Q Qian Z Huang Y Yang X Yang J Xiao N . Mechanisms of endothelial senescence and vascular aging. Biogerontology. (2025) 26:128. doi: 10.1007/s10522-025-10279-y, 40560245

[27] Liu L Zhao B Yu Y Gao W Liu W Chen L . Vascular aging in ischemic stroke. J Am Heart Assoc. (2024) 13:e033341. doi: 10.1161/JAHA.123.033341, 39023057 PMC11964078

[28] Park JH Ovbiagele B. Neurologic symptom severity after a recent noncardioembolic stroke and recurrent vascular risk. J Stroke Cerebrovasc Dis. (2015) 24:1032–7. doi: 10.1016/j.jstrokecerebrovasdis.2014.12.033, 25817617 PMC4408262

[29] Liu X Zhang T Yang L Chen G Ding P Yu D . National Institute of health stroke scale score mediated the relationship between systemic inflammatory response index, high-sensitivity C-reactive protein, and functional prognosis of acute ischemic stroke: a prospective cross-sectional study. Heart Mind. (2025) 9:174–84. doi: 10.4103/hm.HM-D-24-00163

[30] Li X Ma G Chen H Zhang L He J Liu S . From heart to mind: research advances in the link between coronary artery disease and mental stress-a narrative review. Heart Mind. (2025) 9:328–43. doi: 10.4103/hm.HM-D-24-00068

